# The clinical utility of FDG PET/CT among solid organ transplant recipients suspected of malignancy or infection

**DOI:** 10.1007/s00259-016-3564-5

**Published:** 2016-11-12

**Authors:** Neval E. Wareham, J. D. Lundgren, C. Da Cunha-Bang, F. Gustafsson, M. Iversen, H. H. Johannesen, A. Kjær, A. Rasmussen, H. Sengeløv, S. S. Sørensen, B. M. Fischer

**Affiliations:** 1 Department of Clinical Physiology, Nuclear Medicine & PET, Rigshospitalet, Blegdamsvej 9, 2100 Copenhagen Ø, Denmark; 2 Centre for Health and Infectious Disease Research (CHIP), Department of Infectious Diseases, Section 2100, Rigshospitalet, Blegdamsvej 9, 2100 Copenhagen Ø, Denmark; 3 Department of Haematology, Rigshospitalet, Blegdamsvej 9, 2100 Copenhagen Ø, Denmark; 4 Department of Cardiology, Rigshospitalet, Blegdamsvej 9, 2100 Copenhagen Ø, Denmark; 5 Department of Surgical Gastroenterology, Rigshospitalet, Blegdamsvek 9, 2100 Copenhagen Ø, Denmark; 6 Department of Nephrology, Rigshospitalet, Blegdamsvej 9, 2100 Copenhagen Ø, Denmark

**Keywords:** Solid organ transplantation, PET/CT, Infection, Malignancy, Diagnostic performance

## Abstract

**Purpose:**

Solid organ transplant (SOT) recipients are at high risk of developing infections and malignancies. ^18^F-FDG PET/CT may enable timely detection of these diseases and help to ensure early intervention. We aimed to describe the clinical utility of FDG PET/CT in consecutive, diagnostic unresolved SOT recipients transplanted from January 2004 to May 2015.

**Methods:**

Recipients with a post-transplant FDG PET/CT performed as part of diagnostic work-up were included. Detailed chart reviews were done to extract relevant clinical information and determine the final diagnosis related to the FDG PET/CT. Based on á priori defined criteria and the final diagnosis, results from each scan were classified as true or false, and diagnostic values determined.

**Results:**

Among the 1,814 recipients in the cohort, 145 had an FDG PET/CT performed; 122 under the indication of diagnostically unresolved symptoms with a suspicion of malignancy or infection. The remaining (N = 23) had an FDG PET/CT to follow-up on a known disease or to stage a known malignancy. The 122 recipients underwent a total of 133 FDG PET/CT scans performed for a suspected malignancy (66 %) or an infection (34 %). Sensitivity, specificity, and positive and negative predictive values of the FDG PET/CT in diagnosing these conditions were 97, 84, 87, and 96 %, respectively.

**Conclusion:**

FDG PET/CT is an accurate diagnostic tool for the work-up of diagnostic unresolved SOT recipients suspected of malignancy or infection. The high sensitivity and NPV underlines the potential usefulness of PET/CT for excluding malignancy or focal infections in this often complex clinical situation.

**Electronic supplementary material:**

The online version of this article (doi:10.1007/s00259-016-3564-5) contains supplementary material, which is available to authorized users.

## Introduction

Solid organ transplant (SOT) recipients have a lifetime increased risk of developing complications related to the transplantation. This is mainly due to the lifelong intensive immunosuppressive therapy the patients receive during and after transplantation, which on one hand enables the survival of the graft, but on the other hand hampers the host immunologic surveillance [1–3]. The most severe consequences of the weakened immune system are severe opportunistic infections [4] and development of malignancies [5–9]. Other factors such as the chronic underlying disease leading to the transplantation and higher rates of co-morbidities also increase the risk of these conditions. SOT recipients have a 3–5-fold higher risk of developing cancers compared to the general population and the cancers developed in this population tend to be more aggressive with higher rates of morbidity and mortality as a consequence [10].

To some extent, administration of antibiotics or chemotherapy combined with a reduction in the immunosuppressive treatment can cure these complications. Unfortunately, a reduction of the immunosuppressive therapy can lead to rejection of the graft and is a serious limitation in the management of these patients. A close monitoring and follow-up of transplant recipients is therefore crucial for timely detection and rapid treatment of infections and malignancies [5–9, 11]. Routine microbiological, biochemical, and imaging follow-up programmes are not always sufficient in diagnosing these conditions, and thus more advanced diagnostic tools are necessary.

Imaging with ^18^F-Fluordeoxyglucose (FDG) positron emission tomography/computed tomography (PET/CT) can detect metabolic changes commonly seen in malignant and inflammatory cells and is a widely used tool in the management of oncological patients, which has been used to localize, stage, and evaluate treatment of a broad spectrum of malignant diseases for more than a decade [12–16]. Furthermore, it is increasingly recognized that FDG PET/CT is also valuable in diagnosing and monitoring lymphoproliferative disorders [17–19] and a number of non-oncologic diseases such as aseptic inflammation and infection [20–26].

FDG PET/CT may thus be a helpful tool in the management of SOT recipients. Conversely, the available literature of the role of FDG PET/CT in transplant recipients is limited and based on few cases or specific clinical issues.

Therefore, we initiated a retrospective review of FDG PET/CT after SOT to examine the diagnostic values of FDG PET/CT in detecting and diagnosing infections and cancer among diagnostic unresolved SOT.

## Materials and Methods

### Study design and participants

In this retrospective cohort study we enrolled all children and adults consecutively transplanted with a heart, lung, liver, or kidney at the Copenhagen University Hospital, Rigshospitalet between January 2004 and May 2015. All patients in this period are registered and followed in an ongoing database: the Management of Post-Transplant Infections in Collaborating Hospitals (MATCH) programme [27]. This includes all liver and lung transplantations in Denmark in that period and all kidney and heart transplantations in the eastern region of Denmark. Eligible recipients were those with an FDG PET/CT performed in the course after transplantation under the indication of suspected infection or malignancy not revealed by routine microbiological, biochemical, or imaging tests.

**Fig. 1:**
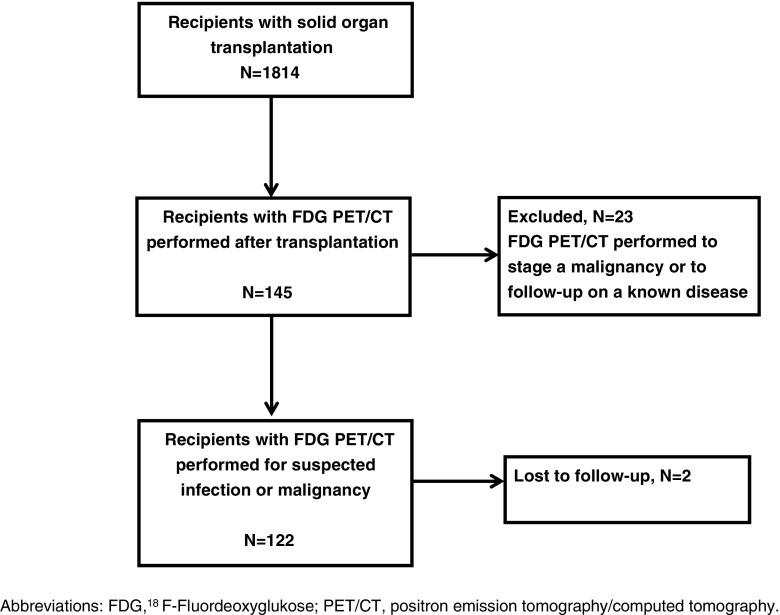
Flow chart of solid organ transplant recipients with a subsequent FDG PET/CT scan; FDG PET/CT performed in diagnostic unresolved recipients suspected of infection or malignancy was included in the study

Recipients where FDG PET/CT was performed to follow-up on an already diagnosed disease were excluded, e.g. surveillance after coincidental finding of a cholangiocarcinoma from the removed liver in a liver transplant recipient, staging of a known lung cancer or follow-up after treatment of an abscess. All referrals were reviewed manually. Patient flow is described in Fig. 1.

The research is conducted after approval of the National Data Protection Agency (2012-58-0004, RH-2015-67, with I-Suite number: 03787) and the Regional Ethics committee (H-2-2014-050).

### FDG PET/CT imaging

All patients were scanned on an integrated FDG PET/CT scanner Biograph TruePoint (16-, 40-, or 64-slice), Siemens Medical Solution, Malvern PA; Biograph 64 mCT, Siemens Medical Solutions, Malvern PA or Discovery LS, 4 Slice, General Electric, Milwaukee, WI, USA). The patients were instructed to fast for at least 6 h before intravenous administration of FDG. A dosage of 200–555 MBq FDG (4 MBq/kg) was injected and the scanning was performed after 60-min rest. A whole body FDG PET/CT was performed (skull base to proximal thigh). The diagnostic CT scans were acquired at 120–140 keV with iodine based intravenous contrast agent unless contraindicated. A multi-bed PET scan, with a scan time of 2–3 min per bed position depending on scanner type and patient BMI, was performed after the CT. CT data were used for attenuation correction. The attenuation corrected PET data were reconstructed iteratively using a 3 D ordered-subset expectation-maximization algorithm (OSEM), for scans performed on the Biograph mCT this included point spread function and time of flight information. For clinical use, all fused PET/CT scans were reviewed by a nuclear medicine physician and a radiologist.

### Follow-up and classification

As part of the study, a detailed chart review was done to confirm disease status and extract additional important clinical details, including lab tests, microbiology, imaging, and pathology, for a period from the first PET/CT scan until death or censoring (minimum 12 months follow-up).

Based on an evaluation of all available data the final clinical diagnosis was determined. Furthermore, in order to evaluate the full spectrum of diagnostic examinations performed prior to the FDG PET/CT, all laboratory data, imaging, biopsies, and culture specimens in the 30 days prior to FDG PET/CT were registered.

A nuclear medicine specialist reviewed the PET/CT reports (blinded to other examinations and clinical follow-up) and classified each scan as normal, suggestive of infection or malignancy, inconclusive, and/or other clinical relevant findings. Furthermore Maximum Standardized Uptake Value normalized to body weight (SUVmax) was registered retrospectively for all lesions described as positive in the PET/CT report (max. five lesions per patient). The results of the FDG PET/CT reports were compared with the final clinical diagnosis by two independent physicians and based on á priori defined classification (see Table 1) each case was then classified as true positive, contributory to diagnosis; true negative, contributory to exclusion; false positive, non-contributory; or false negative, non-contributory.

**Table 1:** Classification of each scan according to the clinical presentation, the clinical FDG PET/CT reports, other available examinations, and treatment response

Classification	Conclusion of the FDG PET/CT	Histology or cytology examination of the abnormality found on the PET/CT or a positive/negative culture	If no histology is available, then all of the following criteria must be in accordance with the PET/CT
True positive, contributory to diagnosis	Abnormality suggestive of infection or malignancy found	Agree with the PET/CT	a) relevant symptoms b) relevant treatment applied and response seen c) no other findings contradicting the results of the PET/CT scan within 3 months
True negative, contributory to exclusion	No abnormality found	Agree with the PET/CT	a) no further symptoms indicating disease developed b) recovery of symptoms without treatment c) no further findings indicating disease within 3 months
False positive, non-contributory	Abnormality suggestive of infection or malignancy found	Disagree with the PET/CT	a) no further symptoms indicating disease developed b) recovery of symptoms without treatment c) no further findings indicating disease within 3 months
False negative, non-contributory	No abnormality found	Disagree with the PET/CT	a) relevant symptoms b) relevant treatment applied and response seen c) further findings contradicting the result of the PET/CT scan within 3 months

Abbreviations: FDG, ^18^F-Fluordeoxyglucose; PET/CT, positron emission tomography/computed tomography

In cases of disagreement an arbitration process among the two reviewers was initiated in order to achieve agreement. In cases with insufficient clinical information and where no certain diagnosis was made, a suggested classification based on the available information was made for the purpose of sensitivity analyses.

### Statistics

Statistical analyses were performed using Statistical Package for the Social Sciences (SPSS) version 22 (IBM, New York, NY, USA). Differences in subgroups were calculated using the Pearson’s Chi-squared test. Sensitivity was defined as: [number of true positive cases]/[total number of true positive and false negative cases]. Specificity was defined as: [number of true negative cases]/[total number of false positive and true negative cases]. Positive predictive value (PPV) was defined as: [number of true positive cases]/[total number of true positive and false positive cases]. Negative predictive value (NPV) was defined as: [number of true negative cases]/[total number of true negative and false negative cases]. Exact 95 % confidence intervals (CI) based on the binomial distribution were calculated for each of these. Cases with no certain diagnosis and where a classification in true or false was not possible were excluded from the analysis initially. A suggested classification was subsequently included in a sensitivity analysis.

## Results

### Patient characteristics

Among the 1814 SOT recipients in the cohort with a median follow-up of 33 months [interquartile range (IQR) 10–69], 145 (8 %) recipients had a total of 219 FDG PET/CT scans performed.

Of those, 122 (84 %) recipients were diagnostically unresolved at time of FDG PET/CT and had a total of 133 FDG PET/CT scans performed under this indication. Twenty-three recipients had a total of 86 FDG PET/CT scans performed for staging and follow-up of known infectious or malignant disease and were excluded from this study, see Fig. 1.

The median time from transplantation to the first FDG PET/CT scan was 19 (IQR 4–49) months. Eleven recipients had a second FDG PET/CT performed a median of 11 (IQR 5–44) months after the first one, due to a new clinical situation without relation to the clinical situation leading to the first FDG PET/CT scan.

Patient characteristics for patients with and without a post-transplant FDG PET/CT scan performed for diagnostic purposes are listed in Table 2. There was no difference between the two groups in terms of gender, age, and time period of transplantation. The proportion of liver and lung transplants was larger among those with a FDG PET/CT compared to those without (38 % vs. 25 % and 26 % vs. 18 % p = 0.002, respectively), whereas the proportion of kidney transplants was smaller (33 % vs. 48 %, p = 0.002).

**Table 2:** Patient Characteristics of recipients with and without a post-transplant FDG PET/CT scan performed for suspected infection or malignancy

Patient characteristics	All recipients	Recipients with a PET/CT N (%)	Recipients with no PET/CT N (%)	p
Total	1814	122 (7)	1692 (93)	-
Gender				0.6
Males	1063 (59)	69 (57)	994 (58)	
Females	751 (41)	53 (43)	698 (42)	
Age at transplantation				0.5
Median,( IQR)	48 (35–57)	49 (34–59)	48 (35–57)	
Type of transplantation				0.002
Kidney	846 (47)	40 (33)	806 (48)	
Liver	493 (27)	46 (38)	447 (26)	
Lung	333 (18)	31 (25)	302 (18)	
Heart	142 (9)	5 (4)	137 (8)	
Year of transplantation				0.8
≤2006	403 (22)	25 (21)	378 (22)	
2007-2009	441 (24)	31 (25)	410 (24)	
2010-2011	374 (21)	29 (24)	345 (20)	
>2011	596 (33)	37 (30)	559 (33)	
Number of PET/CT scans performed				-
1	111 (91)	111 (91)	0 (0)	
2	11 (9)	11 (9)	0 (0)	

Abbreviations: FDG ^18^F-Fluordeoxyglucose, PET/CT positron emission tomography/computed tomography, IQR interquartile range, N number

The first FDG PET/CT scan was performed due to suspected malignancy in 80/122 or infection in 42/122 recipients, whereas the 11 recipients with a second FDG PET/CT had this performed due to suspected malignancy in 8/11 or infection in 3/11. At time of the FDG PET/CT (N = 133), the recipients presented with fever of unknown origin (FUO) in 38 of 133, organ specific symptoms such as diarrhoea, stomach pain, coughing, and neurologic symptoms in 39 of 133, one or more B-symptoms in 25 of 133, and altered biochemical or microbial markers such as sustained elevated CRP, LDH, ALT, or Epstein-Barr virus (EBV) polymerase chain reaction (PCR) in 30 of 133. In one recipient, the FDG PET/CT was performed due to coincidental finding of pathologic appearing lymph nodes of the liver hilum on a CT scan. The patients were examined with other diagnostic procedures within 30 days from FDG PET/CT in 132/133 (99 %) of the cases. This included laboratory analysis in 118/133 (89 %), culture samples in 101/133 (76 %), other imaging in 96/133 (72 %), or biopsies in 54/133 (41 %), respectively. See Online Resource 1 for further details on type and frequency of diagnostic analysis prior to FDG PET/CT. One case suspected for occult cancer due to weight loss and loss of appetite over a period of a few months, only blood samples were taken 43 days prior to the FDG PET/CT.

### The final clinical diagnosis in relation to the FDG PET/CT (N = 133)

**Fig. 2:**
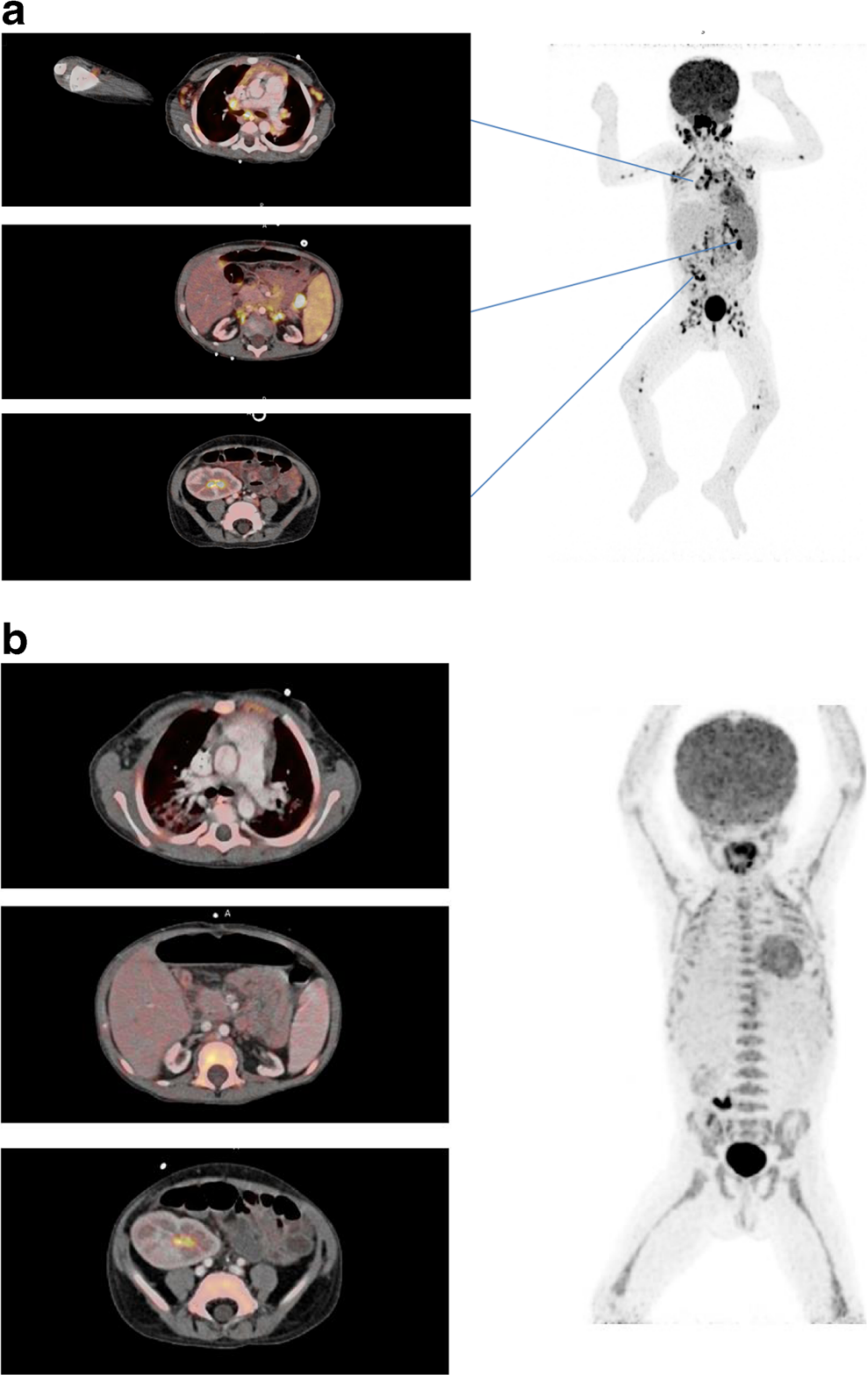
A three year old kidney and liver recipient with fever and elevated EBV DNA in plasma 3 months after transplantation, suspected for post-transplant lymphoproliferative disorders (PTLD). A) The FDG PET/CT showed increased FDG uptake in enlarged and normal sized lymph nodes above and below diaphragm including the extremities, in the rhinopharynx, tonsils, and spleen compatible with post-transplant lymphoproliferative disorders. B) Complete metabolic and structural remission after 3 weeks of rituximab treatment. The area with increased FDG-retention in the right fossa corresponds to urine excretion in the well-functioning graft

The complete diagnostic work-up lead to no pathology in 41 of 133 (31 %), a cancer diagnosis in 32 of 133 (24 %) [including post-transplant lymphoproliferative disorders (PTLD) (N = 12, Fig. 2), lung cancer (N = 7), liver cancer (N = 3), metastasis with unknown primary tumour (N = 2), acute myeloid leukaemia (AML) (N = 1), angiosarcoma (N = 1), bladder cancer (N = 1), cervical cancer (N = 1), gall bladder cancer (N = 1), renal cancer (N = 1), prostate cancer (N = 1), non-melanoma skin cancer (N = 1)], an infection diagnosis in 32 of 133 (24 %) (including 24 focal and eight disseminated infections), and other specific findings of potential clinical relevance in 18 of 133 (14 %), such as rejection of the liver or lung graft or unspecific inflammation of a lymph node.

In ten cases (7 %) determination of a final diagnosis was not possible, as the patients were lost to follow up (N = 2) or due to lack of sufficient clinical and diagnostic information (N = 8). The latter are described in details in Table 3.

**Table 3:** Diagnostic unresolved recipients where classification of the FDG PET/CT scan as true or false was not possible due to lack of sufficient clinical and diagnostic information, and a suggested classification for the sensitivity analysis

Cases	Type of transplant	Symptoms/clinical findings	Suspected condition	FDG PET/CT finding	Conclusion	Suggested classification
1	Liver	Fatigue Hyperbilirubinemia Lymphopenia	PTLD	Inflammation of the lung and porta hepatica	No explanation for the symptoms was found. Infection, lymphoma and rejection of the graft were ruled out.	True negative
2	Liver	Nightly sweating	Malignancy	Inflammation or infection of lungs, tonsils, and lymph node on neck	Histology examination of lymph node on neck was normal. No further explanation for the symptoms was found. Improved spontaneously without treatment.	False positive
3	Kidney	Fever, consistent elevated CRP despite empiric antibiotic treatment (FUO)	Focal infection or malignancy	Activated bone marrow Inflammation of small intestine	No infections were detected. The patient improved on empiric antibiotic treatment.	True negative
4	Kidney	Haemolytic anaemia	Malignancy	No abnormality	Complicated patient with possible, but non-confirmable myelodysplastic syndrome. Developed endocarditis and sepsis and died from his complications three months after the PET/CT scan was performed. No certain diagnosis was made.	False negative
5	Kidney	Unexplained elevated CRP Normal leukocytes	Infection	No abnormality	Known HIV infected patient. No explanation for the elevated CRP was found.	True negative
6	Kidney	Fever and malaise Elevated CRP and ESRD (FUO)	Infection or malignancy	Inflammation of the lung	No infection was detected. Presumed infection. Improved on continued treatment with empiric antibiotics	True positive
7	Kidney	Vomiting and fever (FUO)	Infection	No abnormality	No infections were detected. Improved on continued empiric antibiotic treatment	True negative
8	Lung	Daily fever independent of empiric antibiotic treatment (FUO)	Infection	No abnormality was found	No infections were detected. Improved on continued empiric antibiotic treatment	True negative

Abbreviations: FDG ^18^F-Fluordeoxyglucose, PET/CT positron emission tomography/computed tomography, PTLD post-transplant lymphoproliferative disorders, FUO fever of unknown origin, HIV human immunodeficiency virus, ESRD end-stage renal disease

### Classification and diagnostic values of the FDG PET/CT scans (N = 133)

The FDG PET/CT suggested a malignancy in 32 (24 %) and an infection in 32 of 133 (24 %), whereas PET/CT were without any suspicion of infection or malignancy in 59 of 133 (44 %) cases. Furthermore, eight patients remained undiagnosed and two were lost to follow-up (8 %).

Classification of the scans as true or false was made in initial agreement by two reviewers in 118 of 133 scans. The remaining 15 were agreed on after an arbitration process.

The scans were classified as true positive in 66 (54 %), true negative in 46 (37 %), false positive in 10 (8 %), and false negative in one (1 %). Sixty-five (53 %) of the scans were confirmed by either microbiology, histology, or cytology examination, whereas the classification was based on patient history and other clinical findings in 58 (47 %), Table 4.

**Table 4:** Classification of FDG PET/CT scans performed in diagnostic unresolved cases suspected of infection or malignancy (N = 123) according to histology examination or clinical history

Classification	Confirmed by histology or cytology examination or a positive/negative culture N (%)	Classification based on patient history, symptoms, treatment, and other findings^1^ N (%)	Total N (%)
True positive, contributory to diagnosis	42 (63)	24 (37)	66 (54)
True negative, contributory to exclusion	12 (26)	34 (74)	46 (37)
False positive, non-contributory	10 (100)	0 (0)	10 (8)
False negative, non-contributory	1 (100)	0 (0)	1 (1)
Total	65 (53)	58 (47)	123 (100)

Abbreviations: FDG ^18^F-Fluordeoxyglucose, PET/CT positron emission tomography/computed tomography, N Number

1. Other findings include findings on other imaging, clinical signs, and treatment response or the absence of all three

In eight cases where no diagnosis could be made initially due to insufficient clinical information, a suggested classification was made for the purpose of sensitivity analysis, Table 3.

The two recipients who were lost to follow-up were excluded from the analysis.

Among the 66 true positive scans, the diagnostic work-up lead to a cancer diagnosis in 19 (29 %), an infection in 28 (42 %), PTLD in 12 (18 %), and other specific findings in seven (11 %) cases.

In eight of ten false positive scans the FDG PET/CT scan suggested malignancy, but this was disproved by histology examination of the malignant appearing area and no malignancy was ever diagnosed during the follow-up period. One FDG PET/CT scan suggested malignancy in the mediastinum and the liver hilum, but histology examinations of both areas rejected malignancy, but suggested rejection of the liver graft and sarcoidosis of the mediastinal lymph nodes. The last false positive scan suggested malignancy in the lungs and the thyroid gland, but fungal infection was detected in bronchoalveolar lavage fluid.

One scan was classified as false negative and was without any focal findings; however, the patient was diagnosed with recurrence of a non-melanoma skin cancer of the cheek, with metastases to the ear, facial nerve and parotid gland within one month from the FDG PET/CT was performed.

The diagnostic values of FDG PET/CT are listed in Table 5.

**Table 5:** Diagnostic values of the 123 FDG PET/CT scans in 122 diagnostic unresolved recipients (123 scans) suspected of infection or malignancy according to diagnosis after complete diagnostic work-up

	All scans (N = 64)	Infection^1^ (N = 32)	Malignancy^2^ (N = 32)
Diagnostic values	% 95 % CI	% 95 % CI^3^	% 95 % CI^3^
Sensitivity	99 (92–100)	100 (87 – 100)	97 (86 – 100)
Specificity	82 (70–91)	-	-
Positive predictive value	87 (77–94)	96 (82 – 100)	100 (91 – 100)
Negative predictive value	98 (89–100)	-	-

Abbreviations: FDG ^18^F-Fluordeoxyglucose, PET/CT positron emission tomography/computed tomography, CI confidence intervals.

1. Diagnostic values of recipients diagnosed with an infection after complete diagnostic work-up.

2. Diagnostic values of recipients diagnosed with a malignancy after complete diagnostic work-up.

3. Specificity and negative predictive values could not be calculated due to few scans in these categories.

Sensitivity analysis including the eight initially unclassifiable scans from Table 3, resulted in sensitivity, specificity, positive predictive and negative predictive values of 97, 84, 87, and 96 %, respectively.

**Fig 3:**
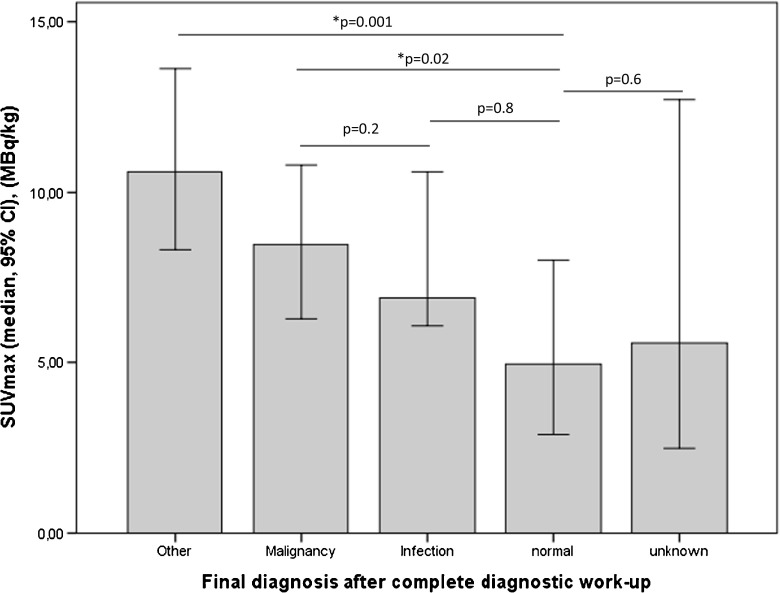
Median maximum standardized uptake values of lesions found on FDG PET/CT (lesion-based analysis, N = 164) according to the final diagnosis

Median SUVmax of findings on FDG PET/CT (N = 164) were lower in cases where follow-up revealed no abnormality compared with those with clinically significant findings (p = 0.03). Comparing PET-positive findings later confirmed as, respectively, malignancy and infection, median SUVmax was lower in the latter, albeit not significantly (p = 0.2), Fig. 3.

## Discussion

This paper presented the diagnostic accuracy of an FDG PET/CT in a large group of SOT recipients with unresolved clinical issues, suspected for a malignancy or an infection. The patients had been examined extensively with other imaging modalities, culture specimens, and other laboratory tests prior to FDG PET/CT without this leading to a diagnosis; however, after an FDG PET/CT scan was performed, cancer or severe infection could correctly be identified or ruled out in the vast majority of the cases (112/123 FDG PET/CT scans) with positive and negative predictive values of 87 and 98 %, respectively. Only in eight cases the recipients remained diagnostic unresolved, even after FDG PET/CT was performed (Table 3). In most of these cases, the symptoms resolved over time in the following couple of months, either spontaneously or on empiric antibiotic treatment, and none were diagnosed with a malignancy or severe focal infection. Our results thus indicate that FDG PET/CT can safely and accurately exclude focal disease in SOT recipients experiencing unexplained symptoms pointing towards malignant or infectious disease. There was a clear tendency towards higher SUVmax of lesions in cases with a clinically significant finding compared to patients without disease (p = 0.03), albeit non-significant when comparing lesions in cases with an infection vs. cancer (p = 0.2). The latter is a well-known drawback of the FDG-PET technology.

Historically, FDG PET/CT has been hampered by a high frequency of false positive findings [23, 28–32], potentially leading to an increasing number of futile invasive tests. In this setting, we found a total of nine false positive malignancies and one false positive infection. In all cases further examinations were made with biopsies and two were diagnosed with an infection and rejection of the graft, respectively. Since the FDG PET/CT could also contribute to the diagnosis of these two it can be debated whether they were in fact false positive. The remaining eight were not diagnosed with any disease in relation to the FDG PET/CT. This relatively low false-positive rate was found despite the application of a rather strict true positive criteria; only FDG PET/CT scans correctly finding and describing changes as either malignancy or infection were classified as true positive. The relatively low number of false positive scans in our study (8 %), compared to previous studies, may likely reflect the á priori high risk of malignancy and infection in this group of patients; in 48 % of the 133 cases either a diagnose of malignancy or infection was confirmed after FDG PET/CT. In other words, unexplained sustained symptoms among SOT recipients, relatively often are an expression of a malignancy or an infection. Furthermore, fever of unknown origin (FUO), which more than one third of the recipients presented with at time of FDG PET/CT has been associated with cancer in up to 30 % of the cases.

FUO usually carries significant diagnostic challenges where no diagnosis can be made in up to 50 % of the patients [24, 33]. FDG PET/CT has been shown to be clinically useful in up to 70 % of these cases with high negative predictive value of up to 100 % [24, 33–36]. The present study confirmed that this also applies for SOT recipients since very few recipients remained diagnostic unresolved after FDG PET/CT was performed.

Only one scan was classified as false negative resulting in a very high negative predictive value.

The false negative scan was found in a recipient with recurrence of a non-melanoma skin cancer of the cheek with spread to the facial nerve and parotid gland. The challenges in discriminating the imaging artefacts related to physiological FDG uptake from those related to malignant uptake is a well-recognized diagnostic dilemma [37], in particular in salivary glands and the nervous system, and may be an explanation for overlooking this cancer case. The high negative predictive value presented here is in accordance with what has been demonstrated in previous studies [21, 24, 26, 38].

The use of FDG PET/CT in the detection and monitoring of graft rejection has been suggested in recent animal based studies [39–42]. None of the scans in the present study were performed for suspected graft rejection and none suggested graft rejection. However, two scans found an abnormal FDG uptake of the graft that was later confirmed to be rejection of the kidney and lung graft, respectively. Three additional recipients were diagnosed with a graft rejection of the lung (N = 1) and liver (N = 2); although these were not detected by the FDG PET/CT scan. The few numbers in this study and the absence of evidence in support of PET/CT in graft rejection stresses the fact that interpretation should be made with caution.

Studies on the clinical value of an FDG PET/CT in suspected infection and inflammation are limited, and it is still debated whether FDG PET/CT actually contributes to the diagnosis or if the diagnosis would have been made independent of the FDG PET/CT. However, with increasing amount of evidence, FDG PET/CT is now recommended in the management of a number of infectious diseases including osteomyelitis, suspected spinal infection, and evaluation of patients with bacteremia [26]. Also, previous studies have demonstrated that early FDG PET/CT in patients suspected for a bacteraemia can ensure timely diagnosis, minimize the admission time of the patients, and improve survival [25, 43].

In the present study, one third of the recipients were diagnosed with a variety of mainly focal infections, and in all but one case, the FDG PET/CT was able to correctly detect the infection. In the one case, the FDG PET/CT suggested malignancy in the lung and thyroid gland, but further examinations revealed fungal infection of the lung. Other cases of fungal infection, for instance, of the lung and liver, were, however, correctly detected by the FDG PET/CT. Thus, FDG PET/CT is likely of clinical value in most cases with focal infections.

Dual time-point (DTP) imaging has been suggested to be able to distinguish malignancies from infection or inflammation and may thus have a potential to help guide clinicians in planning the appropriate intervention. The main rationale being different metabolic rates between malignant and benign cells resulting in different retention index, thus enabling discrimination between increased FDG-uptake on a malignant respectively inflammatory basis [44]. However, more recent studies and meta-analysis have failed to prove a significant clinical benefit of DTP imaging [45–47]. Because of the retrospective nature of our study, it was not possible to assess DTP imaging in SOT patients.

Since FDG may give false positive results due to its unspecific nature towards malignant and inflammatory cells, new imaging tracers specifically targeted towards inflammatory cells are being investigated intensively. PET imaging using tracers such as translocator protein, formyl peptide receptor, and COX inhibitors has showed promising results in regards of detecting neuroinflammation, arteriosclerosis and in inflamed lungs [48]. Further investigations are, however, required before these can be introduced in the clinical setting.

The present study is limited by the fact that it is a retrospective and non-controlled study. We cannot exclude that some patients may have been diagnosed and treated successfully independent of the FDG PET/CT. Neither can we conclude that the FDG PET/CT is in fact better than other diagnostic procedures since we have no control group. However, the general indication for an FDG PET/CT in this cohort was suspicion of an infection or malignancy not revealed by standard diagnostic procedures, and thus most patients had undergone an exhausting diagnostic algorithm prior to the FDG PET/CT. Furthermore, the indications for an FDG PET/CT were unchanged during the entire cohort period and among the different departments.

Since very few studies have examined the role of an FDG PET/CT among transplant recipients a retrospective design was chosen to elucidate this subject. Further studies of prospective nature are recommended to confirm our findings. We have tried to minimize the potential selection bias by including a large cohort of all SOT, transplanted consecutively at a national transplant centre and included in an ongoing database, i.e. the MATCH programme. The cohort is thus likely to represent a non-selected group of patients. We only included recipients with unresolved clinical issues prior to FDG PET/CT, which is likely to represent a more diagnostic challenging group of patients and furthermore, the included and excluded recipients were comparable in regards of demographics and calendar period of transplantation.

To our knowledge this is the first study to describe the diagnostic role of FDG PET/CT in non-selected diagnostic unresolved SOT recipients. We have demonstrated that the use of FDG PET/CT in the follow-up of SOT recipients with non-specific and unexplained symptoms at our hospital has high diagnostic values and can reliably detect or exclude malignancies or infections.

## Electronic supplementary material

Below is the link to the electronic supplementary material.

ESM 1 (DOCX 28 kb)

## Abbreviations

AML: Acute Myeloid Leukaemia
CHIP: Centre of Health and Infectious Disease Research
CI: Confidence intervals
CT: Computed tomography
EBV: Epstein-Barr virus
ESRD: End-stage renal disease
FDG: ^18^F-Fluordeoxyglucose
FUO: Fever of unknown origin
HIV: Human immunodeficiency virus
IQR: Interquartile range
KeV: Kiloelectronvolts
MATCH: Management of Post-Transplant Infections in Collaborating Hospitals
MBq: Megabecquerel
NPV: Negative predictive value
PCR: Polymerase chain reaction
PET: Positron emission tomography
PPV: Positive predictive value
PTLD: Post-transplant lymphoproliferative disorders
SOT: Solid organ transplant
SPSS: Statistical Package for the Social Sciences
SUV max: Maximum Standardized Uptake
DTP: Dual time-point
