# Supplementary material for: The clinical utility of FDG PET/CT among solid organ transplant recipients suspected of malignancy or infection
Source: Eur J Nucl Med Mol Imaging. 2016 Nov 12;44(3):421–31. doi: 10.1007/s00259-016-3564-5 (PMC5281676; doi:10.1007/s00259-016-3564-5)
Supplement: Supplementary file 1 — (DOCX 28 kb) [file 259_2016_3564_MOESM1_ESM.docx]

**Full title: The clinical utility of FDG PET/CT among solid organ transplant recipients suspected of malignancy or infection**

**Running title: FDG PET/CT in solid organ recipients**

**Journal: European Journal of Nuclear Medicine & Molecular Imaging**

Wareham NE^2^, Lundgren JD^2^, Da Cunha-Bang C^3^, Gustafsson F^4^, Iversen M^4^, Johannesen HH^1^, Kjær A^1^, Rasmussen A^5^, Sengeløv H^3^, Sørensen SS^6^, Fischer BM^1^

^1^Department of Clinical Physiology, Nuclear Medicine & PET, Rigshospitalet, Copenhagen University Hospital, Copenhagen, Denmark.

**^2^**CHIP, Department of Infectious Diseases, Rigshospitalet, Copenhagen University Hospital, Copenhagen, Denmark.

^3^ Department of Haematology, Rigshospitalet, Copenhagen University Hospital, Copenhagen, Denmark.

^4^Department of Cardiology, Rigshospitalet, Copenhagen University Hospital, Copenhagen, Denmark.

^5^Department of Surgical Gastroenterology, Rigshospitalet, Copenhagen University Hospital, Copenhagen, Denmark.

^6^Department of Nephrology, Rigshospitalet, Copenhagen University Hospital, Copenhagen, Denmark.

**Corresponding author**

Neval E. Wareham, MD

Rigshospitalet, University of Copenhagen

CHIP, Department of Infectious Diseases, Section 2100

Finsencentret

Blegdamsvej 9

2100 Copenhagen Ø, Denmark

E-mail: neval.ete.wareham@regionh.dk

Phone: +45 35 45 57 97

Fax: +45 34 45 40 15

**Online Resource 1. Type and number of diagnostic tests preceding an FDG PET/CT according to classification of the FDG PET/CT.**

|  | All FDG PET/CT  (N=133) | True positive  (N=66) | True Negative  (N=46) | False Positive  (N=10) | False Negative  (N=1) | Unknown or lost to follow-up  (N=10) | P |
| --- | --- | --- | --- | --- | --- | --- | --- |
| Other imaging^1^  US  N (%)  Median (IQR)  X-ray  N (%)  Median (IQR)  CT  N (%)  Median (IQR)  MRI  N (%)  Median (IQR)  Other^2^  N (%)  Median (IQR) | **46 (35)**  **2 (1-2)**  **71 (53)**  **2 (1-4)**  **47 (35)**  **1 (1-1)**  **10 (8)**  **1 (1-1)**  **15 (11)**  **1 (1-2)** | **27 (41)**  **2 (1-3)**  **40 (61)**  **2 (1-6)**  **31 (47)**  **1 (1-1)**  **5 (8)**  **1 (1-1)**  **5 (15)**  **1 (1-3)** | **15 (32)**  **2 (1-2)**  **21 (46)**  **2 (2-4)**  **11 (24)**  **1 (1-1)**  **4 (9)**  **1 (1-2)**  **5 (6)**  **1 (1-2)** | **2 (20)**  **2 (1-)**  **5 (50)**  **1 (1-2)**  **3 (30)**  **2 (1-)**  **1 (10)**  **1 (1-1)**  **3 (6)**  **1 (1-1)** | **0 (0)**  **-**  **0 (0)**  **-**  **0 (0)**  **-**  **0 (0)**  **-**  **0 (0)**  **-** | **2 (20)**  **2 (1-)**  **5 (50)**  **2 (1-3)**  **2 (20)**  **1 (1-1)**  **0 (0)**  **-**  **2 (20)**  **1 (1-)** | **0.5**  **0.4**  **0.4**  **0.17**  **0.08**  **0.27**  **0.9**  **0.4**  **0.3**  **0.5** |
| Biopsy (histology or cytology)^3^  N (%)  Median (IQR) | **54 (41)**  **1 (1-2)** | **27 (41)**  **1 (1-2)** | **14 (30)**  **1 (1-2)** | **6 (60)**  **1 (1-2)** | **1 (100)**  **1 (1-1)** | **6 (60)**  **1 (1-2)** | **0.2**  **0.9** |
| Culture^4^  Blood culture  N (%)  Median (IQR)  Urine culture  N (%)  Median (IQR)  Culture, other^5^  N (%)  Median (IQR)  Culture faeces  N (%)  Median (IQR)  Culture respiratory secretions  N (%)  Median (IQR) | **82 (62)**  **2 (1-4)**  **62 (47)**  **2 (1-3)**  **45 (34)**  **1 (1-3)**  **38 (29)**  **2 (1-2)**  **29 (22)**  **1 (1-3)** | **42 (64)**  **2 (1-5)**  **32 (48)**  **2 (1-4)**  **31 (47)**  **2 (1-3)**  **20 (30)**  **2 (1-4)**  **20 (30)**  **1 (1-3)** | **30 (65)**  **2 (1-3)**  **20 (63)**  **2 (1-3)**  **11 (24)**  **1 (1-4)**    **11 (24)**  **1 (1-2)**  **6 (13)**  **1 (1-2)** | **4 (40)**  **4 (3-6)**  **4 (40)**  **2 (1-4)**  **2 (20)**  **3 (1-)**  **4 (40)**  **3 (1-5)**  **2 (20)**  **1 (1-)** | **0 (0)**  **-**  **0 (0)**  **-**  **0 (0)**  **-**  **0 (0)**  **-**  **0 (0)**  **-** | **6 (60)**  **1 (1-6)**  **6 (60)**  **3 (1-3)**  **1 (10)**  **3 (3-3)**  **3 (30)**  **1 (1-)**  **1 (10)**  **1 (1-1)** | **0.4**  **0.3**  **0.7**  **0.7**  **0.06**  **0.2**  **0.8**  **0.1**  **0.2**  **0.6** |
| Laboratory tests^6^  CRP (mg/L)  N (%)  Median (IQR)  Leukocyte count (x10^9^/L)  N (%)  Median (IQR)  Hb (mmol/L)  N (%)  Median (IQR)  Na (mmol/L)  N (%)  Median (IQR)  K (mmol/L)  N (%)  Median (IQR)  ALT (U/L)  N (%)  Median (IQR)  Bilirubine (μmol/L)  N (%)  Median (IQR)  Creatinine (μmol/L)  N (%)  Median (IQR)  LDH  N (%)  Median (IQR) | **117 (95)**  **9 (3-20)**  **117 (95)**  **9 (3-20)**  **118 (97)**  **9 (3-20)**  **118 (97)**  **9 (3-19)**  **118 (97)**  **9 (3-19)**  **112 (92)**  **6 (2-14)**  **111 (91)**  **6 (2-14)**  **118 (97)**  **9 (2-19)**  **112 (92)**  **5 (2-16)** | **62 (94)**  **15 (4-21)**  **62 (94)**  **15 (4-23)**  **63 (95)**  **12 (3-21)**  **63 (95)**  **14 (3-23)**  **63 (95)**  **14 (3-23)**  **63 (95)**  **6 (2-15)**  **62 (94)**  **7 (2-15)**  **63 (94)**  **12 (3-21)**  **60 (91)**  **7 (3-17)** | **40 (87)**  **4 (2-14)**  **40 (87)**  **4 (2-14)**  **40 (87)**  **5 (2-15)**  **40 (87)**  **4 (2-14)**  **40 (87)**  **4 (2-14)**  **36 (78)**  **4 (2-7)**  **36 (78)**  **4 (2-9)**  **40 (87)**  **4 (2-15)**  **37 (80)**  **3 (2-8)** | **7 (70)**  **12 (4-21)**  **7 (70)**  **12 (4-21)**  **7 (70)**  **12 (4-21)**  **7 (70)**  **12 (4-21)**  **7 (70)**  **12 (4-21)**  **7 (70)**  **10 (4-13)**  **7 (70)**  **10 (4-15)**  **7 (70)**  **12 (5-21)**  **7 (70)**  **12 (4-21)** | **0 (0)**  **-**  **0 (0)**  **-**  **0 (0)**  **-**  **0 (0)**  **-**  **0 (0)**  **-**  **0 (0)**  **-**  **0 (0)**  **-**  **0 (0)**  **-**  **0 (0)**  **-** | **8 (80)**  **9 (5-22)**  **8 (80)**  **9 (5-22)**  **8 (80)**  **9 (5-22)**  **8 (80)**  **9 (3-22)**  **8 (80)**  **9 (3-22)**  **6 (60)**  **4 (3-9)**  **6 (60)**  **5 (3-9)**  **8 (80)**  **9 (3-23)**  **8 (80)**  **9 (2-20)** | **0.01**  **0.2**  **0.01**  **0.2**  **0.004**  **0.2**  **0.004**  **0.3**  **0.004**  **0.3**  **0.001**  **0.2**  **0.002**  **0.1**  **0.004**  **0.2**  **0.05**  **0.1** |

Abbreviations: US, ultra sound; X-ray,x-radiations; CT, computed tomography; MRI, magnetic resonance imaging; CRP, c reactive protein; Hb, hemoglobine; Na, sodium; K, potassium; ALT, alanine aminotransferase; LDH, lactate dehydrogenase.

^1^ Number of patients with at least one other imaging prior to FDG PET/CT and median number of imaging tests performed per patient.

^2^ Includes imaging such as scintigraphy, pyelography and cholangiography.

^3^ Number of patients with at least one biopsy prior to FDG PET/CT and median number of biopsies performed per patient.

^4^ Number of patients with at least one culture specimen prior to FDG PET/CT and median number of cultures performed per patient.

^5^ Includes joint fluid, skin specimens, ascites fluid, pleural fluid, spinal fluid.

^6^ Number of patients with at least one laboratory test prior to FDG PET/CT and median number of tests performed per patient. Laboratory data were available from 2010 and thus collected for 118/133 FDG PET scan cases; 63/66 True Positive; 40/46 True Negative; 7/10 False Positive; 8/10 with unknown disease.
